# Recruiting In Vitro Transcribed mRNA against Cancer Immunotherapy: A Contemporary Appraisal of the Current Landscape

**DOI:** 10.3390/cimb45110576

**Published:** 2023-11-16

**Authors:** Androulla N. Miliotou, Sofia K. Georgiou-Siafis, Charikleia Ntenti, Ioannis S. Pappas, Lefkothea C. Papadopoulou

**Affiliations:** 1 Laboratory of Pharmacology, School of Pharmacy, Faculty of Health Sciences, Aristotle University of Thessaloniki, 54124 Thessaloniki, Macedonia, Greece; amiliotou@pharm.auth.gr (A.N.M.); sgeorgi@pharm.auth.gr (S.K.G.-S.); charnten@auth.gr (C.N.); 2 Department of Health Sciences, KES College, 1055 Nicosia, Cyprus; 3 Faculty of Pharmacy, Department of Health Sciences, University of Nicosia, 1700 Nicosia, Cyprus; 4 Laboratory of Pharmacology and Toxicology, Faculty of Veterinary Medicine, University of Thessaly, 43100 Karditsa, Thessaly, Greece; ipappas@vet.uth.gr; 5 1st Laboratory of Pharmacology, School of Medicine, Faculty of Health Sciences, Aristotle University of Thessaloniki, 54124 Thessaloniki, Macedonia, Greece

**Keywords:** IVT-mRNA, cancer immunotherapy, IVT-mRNA cancer vaccines, personalized immunotherapy, Chimeric Antigen Receptor (CAR), CAR T-cell therapy, Protein Transduction Domain, PTD-IVT-mRNA, CAR NK-92 cells, bioenergetics

## Abstract

Over 100 innovative in vitro transcribed (IVT)-mRNAs are presently undergoing clinical trials, with a projected substantial impact on the pharmaceutical market in the near future. Τhe idea behind this is that after the successful cellular internalization of IVT-mRNAs, they are subsequently translated into proteins with therapeutic or prophylactic relevance. Simultaneously, cancer immunotherapy employs diverse strategies to mobilize the immune system in the battle against cancer. Therefore, in this review, the fundamental principles of IVT-mRNA to its recruitment in cancer immunotherapy, are discussed and analyzed. More specifically, this review paper focuses on the development of mRNA vaccines, the exploitation of neoantigens, as well as Chimeric Antigen Receptor (CAR) T-Cells, showcasing their clinical applications and the ongoing trials for the development of next-generation immunotherapeutics. Furthermore, this study investigates the synergistic potential of combining the CAR immunotherapy and the IVT-mRNAs by introducing our research group novel, patented delivery method that utilizes the Protein Transduction Domain (PTD) technology to transduce the IVT-mRNAs encoding the CAR of interest into the Natural Killer (NK)-92 cells, highlighting the potential for enhancing the CAR NK cell potency, efficiency, and bioenergetics. While IVT-mRNA technology brings exciting progress to cancer immunotherapy, several challenges and limitations must be acknowledged, such as safety, toxicity, and delivery issues. This comprehensive exploration of IVT-mRNA technology, in line with its applications in cancer therapeutics, offers valuable insights into the opportunities and challenges in the evolving landscape of cancer immunotherapy, setting the stage for future advancements in the field.

## 1. IVT-mRNA: A Comprehensive Exploration of the Technology

The field of RNA research has been expanding since RNA was identified as a molecule, independent of DNA, in the 1930s. Since 1957, 31 Nobel Prizes have been awarded for RNA biology, with nine Nobel Prizes in Chemistry and 22 Nobel Prizes in Physiology or Medicine, including the one awarded at the beginning of October 2023, to Katalin Karikó and Drew Weissman for their discoveries that enabled the development of the mRNA vaccines against SARS-CoV-2 during the COVID-19 pandemic. RNA-based therapies have tremendous potential to treat many diseases that currently lack or have no optimal treatment options [1]. In fact, according to Allied Market Research estimates, the RNA-based therapy sector is expected to reach a valuation of $25 billion by 2030 [2].

The basic idea behind in vitro transcribed (IVT)-mRNA therapies is to employ mRNA, synthesized in vitro, to induce the translation of proteins that have pharmacological action in vivo. Since it is encoded by IVT-mRNA, theoretically, any functional protein or peptide may be produced-using this method-for protein replacement therapy or immunization. However, since the discovery of mRNA 60 years ago, it has taken scientists many years and significant effort to effectively generate a final, efficient and commercial product. Precisely, IVT-mRNA has come a long way, with the recent approval of two COVID-19 mRNA-based vaccines (BNT162b2 and mRNA-1273), to finally show its immense potential. Its numerous uses have made it possible to develop treatments for a wide range of diseases, and in recent years, the number of clinical studies has exhibited a tremendous increase. 

IVT-mRNA chemical alterations and the development of delivery vehicles, as well as its characteristics and therapeutic goals, are crucial for ensuring clinical success. The COVID-19 vaccines, the first mRNA-based pharmaceuticals to be authorized and distributed globally, as well as cancer immunotherapy, are setting the standard for the therapeutic application of mRNA. Although it is a novel therapeutic strategy that may be used to treat a wide range of diseases and there are still many issues that arise for its exploitation, however, IVT-mRNA is here to stay [3]. 

IVT-mRNA-based technology is currently thought to fill a discrete gap between gene therapy and protein therapy. Compared to the other two macromolecule approaches, it demonstrates a number of outstanding benefits. Therapeutics based on IVT-mRNA have significant advantages over recombinant proteins, since post-translational modifications, folding, and localization of exogenously generated proteins are obstacles that IVT-mRNA overcomes. In the meantime, it may simultaneously encode numerous proteins for immunogens (made up of many subunits), which overcomes the difficulty of reassembling the protein subunits with the right stoichiometry [4,5]. The design methodology for IVT-mRNA brings notable versatility, as it allows the same platform to be used for various targets. Additionally, IVT-mRNA can be rapidly designed, and large-scaled generated, it is highly adaptable, and less costly than those of proteins. On the other hand, unlike DNA therapeutics, IVT-mRNAs do not need to enter the nucleus in order to function, meaning that the risk of insertional mutagenesis is eliminated [6,7]. Furthermore, IVT-mRNA is a transiently active molecule, leading to minimal homeostasis destruction, compared to the permanent nature of DNA-based approaches. These outstanding benefits make IVT-mRNA technology the most recent and promising therapeutic approach, attracting a lot of interest from the scientific community, funding organizations, and the biomedical sector.

Embarking from the beginning, during the year 1961, mRNA was introduced to the scientific community by two Nature scientific papers, by Brenner et al. and Gros et al. [8,9], and a review paper by Jacob and Monod [10], and since then, the role of mRNA has been explored, either for activating or blocking protein expression in the context of therapeutics [11]. When a fledgling San Diego biotech company, named Vical Incorporated, announced its initial results in 1989, the idea of using mRNA as a potential therapeutic drug took off. They showed that different eukaryotic cells may effectively be transfected by mRNA contained within a liposomal nanoparticle. Another crucial finding on the path to mRNA vaccines was the study of Malone et al. in 1989, showing that exogenous luciferase-coding mRNA could be introduced into a cell line via liposomes, leading to enzyme production [12]. A few months later, Wolff et al. published the results of their studies in which mice were directly intramuscularly (i.m.) administered with naked mRNA (firstly serving as a control to a lipid-mediated delivery platform), leading to the production of the encoded protein over the course of a few days [13]. IVT-mRNA was first used in 1992 by Jirikowski et al. to treat diabetes in mice [14]. In 1995, the first IVT-mRNA vaccination for cancer was applied [15]. In 1996, Gilboa and team proposed the use of IVT-mRNA vaccines in patients with microscopic tumors [16]. In the late 1990s, the team of Hoerr and colleagues discovered that using intradermal injection of IVT-mRNA, skin cells can express the corresponding proteins encoded by the mRNA, and in parallel, the common belief at the time that IVT-mRNA was unstable was refuted [17]. In fact, mRNA must overcome many obstacles to be successfully delivered into target cells, since evolutionary barriers exist to block intracellular delivery of foreign RNA. Following in vivo administration, mRNA must also overcome the degradation by harmful RNases present in the tissues and bloodstream. Furthermore, mRNAs are large and polyanionic molecules; hence passive diffusion across the cell membrane is not efficient. Therefore, mRNA molecules rely mainly on active endocytic uptake mechanisms, which may lead to entrapment and degradation in the endosome [18]. Moreover, immunization can be initiated with a minimal protein production because the immune system has the capacity to greatly enhance the antigenic signal through both cell-mediated and antibody-mediated immunity. On the other hand, mRNA therapeutics necessitate a significantly higher protein production, often up to a thousand times more, to reach the required therapeutic threshold [19].

Many viruses contain RNA genomes, while the immune system is extremely sensitive to RNA, and its presence inside cells can be a symptom of viral infection. Toll-like receptors, which are involved in host immunological recognition of RNA, cause cellular responses that can block protein synthesis and destroy nucleic acids. Therefore, to avoid inducing an immune response, it was necessary to figure out a technique to go around the immune system’s RNA surveillance systems, to facilitate IVT-mRNA therapeutic function. 

Katalin Karikó and Drew Weissman were laureated the 2023 Nobel Prize in Physiology or Medicine for their pioneering research on IVT-mRNA, conducted at the University of Pennsylvania, demonstrating the possibility of reducing the body’s inflammatory responses to IVT-mRNAs. This breakthrough paved the way for the development of mRNA-based vaccines, including those that protect against COVID-19 [20]. In their study, ex vivo human Dendritic Cells (DCs) were exposed to mRNA derived from several sources. DCs tolerated mammalian mRNA, but not mRNA from bacteria and necrotic mammalian cells, nor IVT-mRNA, which caused severe inflammatory cytokine reactions [21]. Interestingly, they discovered that the presence of modified nucleotides in the IVT-mRNA construct, such as methylated nucleosides or pseudouridine (Ψ), might be responsible for the drastically diminished immune-modulatory potential of the exogenous mRNA. Thus, it was determined that modified nucleotides shield IVT-mRNA from immune recognition and enable cells to distinguish it from pathogenic or foreign mRNA [21]. In order to increase cell survival and protein synthesis, as well as to reduce immunogenicity, methylpseudouridine (m1Ψ) in conjunction with 5-methylcytidine (5mC) was also suggested to be incorporated in IVT-mRNA synthesis [22,23]. Over the subsequent years, Karikó’s and Weissman’s work evolved, showing in 2008 that Ψ could increase protein production in cells that absorbed the modified mRNA. They also uncovered the cellular mechanisms behind these findings [24,25].

Additionally, numerous cellular activities (including translation, splicing, and destruction) heavily rely on the mRNA 5′ cap structure. More than half of the caps were usually being put inverted during the transcription of the IVT-mRNA (using a bacteriophage promoter), rendering them invisible to the mRNA-stabilizing cap-binding proteins. In order to solve this difficulty, to increase translational capacity and enhance capping efficiency, anti-reverse cap analogs (ARCAs), structured 3′-O-Me-m7G(5)ppp(5)G, were developed. Methoxy group was used to either substitute or omit the typical 3′-OH of the natural cap to prevent misalignment [26]. Moreover, the 3′-untranslated regions (UTRs) of most eukaryotic mRNAs contain Adenylate-Uridylate-rich Elements (AREs), which are indicators of mRNA degradation, regulating mRNA output from the nucleus and translation efficiency, as well as subcellular localization and stability. The use of 3′-UTRs of more stable mRNAs, such as those generated from α- or β-globin mRNAs, as well as the addition of a potent Kozak sequence, can boost the IVT-mRNA stability and translation. The IVT-mRNA is also turned into a very stable molecule by the long poly-(A) tail (100–300 nucleotides), since removing it makes the IVT-mRNA more susceptible to degradation. Another IVT-mRNA optimization strategy, known as codon optimization, leverages the more common codons in the open reading frame of the IVT-mRNA to speed up translation without changing the final protein sequence [27]. Zhang et al. presented an algorithm, a part of an mRNA designing tool, aiming to predict the most-stable IVT-mRNA sequence and its respective structure (not including UTRs sections and modified nucleotides), in just 11 min [28].

Advancements in machine learning have also introduced innovative concepts for optimizing the design of IVT-mRNA sequences [29]. Recent progress in bioinformatics has also facilitated the development or adaptation of a diverse set of computational tools tailored for IVT-mRNA design. Among these, machine learning-based epitope prediction models hold significant promise for enhancing mRNA vaccine design. Integrating epitope-based design procedures into the rational vaccine development process for mRNA vaccines have the potential to significantly boost vaccine immunogenicity and practical efficacy [30]. Additionally, the structural analysis of broadly neutralizing antibodies generated in response to SARS-CoV-2 can serve as a guide for designing antigens in mRNA vaccines, with the aim of providing broad protection against multiple viral variants [31]. 

Other approaches, such as self-amplifying mRNA (SAM), have the potential to reduce the need for multiple vaccine doses by significantly increasing antigen expression with smaller amounts. However, as one of the key features of SAM RNA vaccines is their ability to replicate within cells, this could potentially lead to a stronger immune response. Thus, controlling the degree of amplification is critical to prevent excessive or uncontrolled replication, which could pose risks. As SAM vaccines introduce genetic material into cells, a concern about potential off-target effects or unintended gene expression changes was raised [32]. SAM vaccines directed against SARS-CoV-2 exhibited limited reactogenicity during clinical trials. This method may ultimately prove more potent and cost-efficient when compared to non-replicating mRNA (NRM) vaccines [33].

The development of liposome and nanoparticle technologies may have made the most underappreciated contribution to the successful production of the IVT-mRNA technology. In fact, over the years, the focus has started to shift towards the development of efficient and protective delivery vehicles, investigating the use of lipids as envelopes for ensuring the successful delivery of the IVT-mRNA [34]. An efficient delivery vehicle must protect IVT-mRNA from degradation and enable intracellular delivery. Effective delivery to solid organs is still difficult, except for the liver, which may be easily targeted through i.v. administration. Repeated dosage, which is frequently necessary in cases of treatment for chronic disorders, represents another significant barrier [19]. Bangham et al. investigated the ability of phospholipids to self-assemble into structures with double-layered membranes that could enclose cations, which was first demonstrated in a landmark work, published in 1965 [35]. Liposomes were approved as a drug-distribution delivery system in the 1990s [36]. The phospholipid nanoparticles DOTMA (N-[1-(2,3-dioleyloxy)propyl]-N,N,N-trimethyl-ammonium chloride), DOTAP (1,2-dioleoyl-3-trimethyl-ammonium-propane), and DOPE (dioleoyl-phosphatidyl-ethanol-amine) were then applied, offering complex support and providing highly stable structure (saturated lipids) and endosome destabilization (unsaturated lipids, like DSPC and DPPC). Later, stabilizing pegylated lipids were developed, like polyethylene glycol (PEG)-DMG (by Moderna, Cambridge, MA, United States) and ALC-0159 (by BioNTech/Pfizer, Mainz, Germany/New York, United States), creating lipid nanoparticles (LNPs) with a hydrophilic surface, steric hindrance, and «stealth» effect [37,38]. 

On the whole, the emergence of COVID-19 represents a pivotal moment in the history of IVT-mRNA vaccines. With the onset of the COVID-19 pandemic, a significant public health crisis has been caused. SARS-CoV-2 caused more than 700 million infections and 6.9 million fatalities [39]. In 2020, global research endeavors expedited the creation of a SARS-CoV-2 vaccine to curb the pandemic. Leading vaccine candidates from BioNTech/Pfizer (Mainz, Germany/ New York, United States) (BNT162b) and Moderna (Massachusetts, United States) (mRNA-1273) swiftly received emergency authorization from the U.S. Food and Drug Administration (FDA) and were manufactured in record time. Both vaccines are composed of LNP vehicles that contain IVT-mRNA encoding the SARS-CoV-2 spike protein. These LNP constructs simulate the viral replication process without the risk of infection and induce an adaptive immune response targeting SARS-CoV-2. Furthermore, ionizable lipids facilitate nucleic acids complexation and membrane fusion, with the ALC-0315 (BioNTech/Pfizer, Mainz, Germany/New York, NY, United States)) and SM-102 (Moderna, Massachusetts, United States), serving as the outer shells of COVID-19 mRNA vaccines. These vaccines performed exceptionally well in Stage III clinical trials, showing 94–95% efficacy, minimal adverse effects, and high immunogenicity [40]. Consequently, they have been used to immunize billions of people worldwide against COVID-19. Additional “booster” doses have proven necessary to replenish declining levels of neutralizing antibodies, but both BNT162b and mRNA-1273 have significantly reduced the incidence of hospitalizations and fatalities related to COVID-19. Consequently, mRNA vaccines have emerged as the next generation of vaccines for frontline protection against infectious diseases, marking a significant development in the evolution of public health practices [41].

Although LNPs have shown considerable promise in many therapeutic applications for the delivery of IVT-mRNA, they also face several challenges. The presence of serum, the size of the lipoplex, the density of the surface charge, the colloidal stability, the endosomal escape, the various uptake mechanisms, the sensitiveness in degradation, and the absence of cellular and nuclear targeting have all been suggested to play a role in the efficacy of transfection via the cationic, lipid nanocarriers [42,43]. Moreover, regardless of the inclusion of targeting segments, carriers such as polymers or LNPs tend to accumulate in the liver and spleen. Among the many characteristics, LNPs size had the strongest association with immunogenicity. In mice, there is a positive association between size and immunogenicity; as the LNPs size increases (to 100 nm), so does the antibody titer. In nonhuman primates (NHPs), immunogenicity is independent of LNPs size, and a significant immune response is induced at all particle sizes investigated [44]. In fact, LNPs activate multiple inflammatory pathways, inducing IL-1β and IL-6. Designing and developing LNPs is a difficult procedure that needs careful optimization. It can be difficult and time-consuming to achieve the ideal balance of lipids, encapsulation effectiveness, and stability [45]. Furthermore, due to their short half-life, LNPs exhibit in vivo instability, resulting in endosomal escape and clearance by macrophages is held rather than the expected cellular uptake [46]. In addition, the instability of LNPs during storage or in physiological settings could result in premature mRNA release or diminished therapeutic efficacy [47]. The LNPs platform may also deal with issues of stability, complicated storage, and distribution.

Due to the versatility that peptides can provide, peptide-based delivery systems are becoming more popular. The peptides have distinct biological features, such as cell and nuclear surface targeting, high cell permeability efficiency, and low molecular weight when compared to cationic polymers [48]. Peptide-based carriers and hybrids are proposed as intriguing alternatives to the different known non-viral vectors for IVT-mRNA delivery, either by non-covalent or covalent binding. A representative example is the Protein Transduction Domains (PTDs) or Cell Penetrating Peptides (CPPs). Exploiting PTDs for IVT-mRNA delivery, our unique, cutting-edge PTD-IVT-mRNA technology seems promising for being effective in delivering therapeutic IVT-mRNA payloads to target cells. This novel strategy combines the adaptability of peptides with the accuracy of IVT-mRNA, opening a viable path for the development of a next-generation non-viral delivery platform in the area of IVT-mRNA therapeutics. PTD-IVT-mRNAs showed encouraging results for two protein-disease-models, including the mitochondrial disorder fatal infantile cardioencephalomyopathy and COX deficiency (attributed to *SCO2* gene mutations) and β-thalassemia [49], as well as in a Chimeric Antigen Receptor (CAR) Immunotherapy of ErbB(+) solid tumor neoplastic cells [50], which are explained thoroughly in the following chapters.

Prophylactic vaccines against infectious diseases, the protein replacement therapy (PRT), and the therapeutic vaccines against cancer as well the cancer immunotherapy are the main applications of IVT-mRNA technology, with outstanding research being made in these areas. Regarding the prophylactic use of IVT-mRNA for immunization against harmful microorganisms, the main strategy includes an antigen-encoded IVT-mRNA to be translated into the corresponding antigen in vivo, that can activate the host’s immune system against the infectious pathogen. Before COVID-19, several attempts had been made to develop IVT-mRNA prophylactic vaccines to fend against various infectious viruses, such as the influenza virus [51], Ebola virus, Zika virus [52], rabies virus, and HIV, while some of the above have proceeded to clinical evaluation [5]. The second application considers monogenic diseases, where essential proteins are expressed abnormally or not at all, and Protein Replacement Therapy (PRT) attempts to replace those defective proteins. The clinical assessment of IVT-mRNA technology in the context of PRT includes the IVT-mRNA of the vascular endothelial growth factor (VEGF, NCT03370887) and the IVT-mRNA of CFTR (NCT03375047) [53], as well as IVT-mRNAs for myocardial infarction and heart failure (NCT03370887) [54] and for genetic lung disorders [55]. In the preclinical stage, there are studies for hemophilia A (factor VIII deficiency) and hemophilia B (factor IX deficiency) [56]. 

This review emphasizes in the third application category, the recruitment of IVT-mRNA technology against cancer. One of the main features of cancer research for a long time has been using the immune system to combat cancer, as demonstrated by the many immunotherapeutic strategies. The combination of cancer immunotherapy with IVT-mRNA technology has gained attention, amidst the challenges posed by the COVID-19 pandemic, and mRNA vaccines have surfaced to address the demand and boost researchers, academics, and industry to the direction of IVT-mRNA immunotherapy tactics, discussed in the following sections.

## 2. IVT-mRNA Innovations against Cancer: A Leap in Cancer Immunotherapy

According to https://clinicaltrials.gov/ (accessed on 15 September 2023), over 100 clinical trials based on RNA technology are currently underway, for Amyotrophic Lateral Sclerosis, Huntington’s disease, Hereditary Angioedema and other disorders. Currently, there are already 18 clinically approved RNA-based therapies, including the vaccines that made mRNA well-known during the COVID-19 pandemic (BioNTech/Pfizer, Mainz, Germany/New York, United States and Moderna, Massachusetts, United States). RNA-based therapies have already been approved for both Spinal Muscular Atrophy and Duchenne Muscular Dystrophy. 

The first conceptualization of an mRNA vaccine for cancer was made in 1995 [57], and the implementation of the idea was first reported by Boczkowski et al. in 1996, where they examined the feasibility of inducing cytotoxic T-cell (CTL) and tumor immunity by pulsing DCs with tumor-derived RNA. DCs pulsed with in vitro-produced chicken ovalbumin (OVA) RNA were more successful in inducing primary, OVA-specific CTL responses in vitro, than DCs pulsed with OVA protein. Mice immunized with DCs pulsed with tumor-derived RNA showed a substantial reduction in lung metastases in the low immunogenic, highly metastatic B16/F10.9 tumor model [15]. 

In fact, DCs, a type of antigen-presenting cells (APCs), play a vital role in the immune system. Through the processing of antigens, DCs activate the adaptive immune response, processing antigens presented to T-cells (CD8+ or CD4+) through Major Histocompatibility Complex (MHC) molecules (MHC class I or MHC class II molecules). CD8+ T-cells and CD4+ T-cells interact through MHC class I and class II molecules, respectively. Additionally, in order to stimulate an immune response, DCs may process antigens. As a result, DCs are an important target for the ex vivo and in vivo transfection delivery of mRNA vaccines. Ex vivo DC loading is being researched for cancer vaccination to promote cell-mediated immunity [58]. In various stages of clinical studies, DC-based mRNA vaccines against cancer have demonstrated encouraging outcomes [59]. Ary et al. recently developed mRNA-lipid nanocomplexes that trigger potent immune responses to stop the growth of the B16-OVA tumor. In tissue culture and mice, the mRNA vaccination was examined and found to improve mouse survival [60].

Lai et al. showed encouraging results for the delivery of the LNP-IVT-mRNA of IL-12 in *MYC*-oncogene-driven hepatocellular carcinoma, resulting in activation of CD44^+^ immune cells, thus reducing tumor size and increasing survival in mice [61]. Moreover, cKK-E12 IVT-mRNA (trastuzumab mRNA), also delivered through LNPs, exhibited higher serum levels of trastuzumab, compared to the biologic drug trastuzumab (Herceptin^®^), in mice with breast cancer [62]. Islam et al. also showed restoration of prostate cancer tumor-growth inhibition in vivo, via systemic IVT-mRNA of *PTEN* (Phosphatase and Tensin homolog deleted on chromosome ten), delivered by a PEG-coated hybrid cationic lipid-like compound (G0-C14) and poly lactic-co-glycolic acid (PLGA) [63].

mRNA-based vaccination aims to stimulate or activate an efficient anti-tumor immune response, triggered in two different ways. After vaccination, mRNAs first enter the cytoplasm, via endocytosis, join ribosomes in the host cell, and undergo effective translation. The proteasome breaks down the antigens into antigenic peptides in the cytoplasm, and these short antigenic peptides are then presented to CTLs by the MHC. As an alternative pathway, the host cell may release antigenic proteins, which are then captured by DCs, broken down, and presented to helper T-cells and B-cells via MHC. Finally, B-cells produce antibodies that are capable of recognizing antigenic proteins [64].

Patients are being enrolled in several clinical trials, such as NCT04534205, NCT03313778, and NCT04503278, for various mRNA-based cancer vaccine therapy studies in an effort to trigger an mRNA-based anti-tumor response [65]. A few clinical studies involve naked, unformulated mRNA vaccines (in a non-recruiting stage), being administered intradermally or intranodally. The limited number of such clinical studies is interpreted by the sensitivity of the naked mRNA to degradation by harmful, extracellular RNases. Thus, several nanocarriers have been included, such as protamine (RNActive vaccine, CV9104, CV9201, CV9202), cationic lipids (BNT111/112/113/114/115, FixVac), lipid nanoparticles (mRNA-4157), etc.

Recently, results were reported by Breda et al. of successful delivery of mRNA into bone marrow stem cells by intravenous injection, loaded into lipid nanoparticles, facilitating both gene editing and bone marrow transplantation. The ability to modify bone marrow cells in vivo, in a patient’s body without the need for traditional transplantation approaches could hold great promise for a range of genetic disorders [66]. 

## 3. Empowering the Immune System: The IVT-mRNA Vaccines and Neoantigens in Cancer Immunotherapy

Ever since Georg Klein’s pioneering discovery of Tumor-Specific Antigens (TSAs) in 1967 [67], there has been significant research into developing cancer vaccines for therapeutic purposes. Currently, there are numerous cancer vaccines under development [68]. Despite these efforts, achieving clinical success has proven challenging, with only a small fraction of clinical trials showing more than 10% of objective clinical responses. Additionally, a very limited number of trials have demonstrated an overall therapeutic benefit exceeding 25% [69,70,71].

Utilizing short or long peptides, generated from tumor antigens, together with the appropriate adjuvant, was one of the early methods for antigen-specific immunotherapy [72]. Although it is technically possible to synthesize these peptides, immunological responses to peptide vaccines are confined to a small number of individuals, who have the right Human Leukocyte Antigen (HLA) haplotype, which restricts their use to larger patient groups [71]. Additionally, the restricted immunogenicity of recombinant proteins and their difficult, costly, and time-consuming manufacturing for vaccine-grade purposes make this technique difficult. At present, there are only two FDA-approved cancer-related prophylactic subunit vaccines, both of which are against viruses known to be associated with cancer, the human papillomavirus (HPV) and the hepatitis B virus (HBV). The PROVENGE (Sipuleucel-T) for prostate cancer vaccine was the first to be licensed by the FDA, after decades of research [73,74]. Patient’s derived APCs are cultured in vitro with Prostatic Acid Phosphatase (PAP) (present in 95% of prostate cancer cells), as the target antigen, which is taken up and presented. These APCs are re-infused back into the patient to activate the CTLs, thus stimulating the T-cell immunity directed against the target antigen, PAP [75].

IVT-mRNA technology started to be exploited in cancer immunotherapy and vaccine research, with encouraging findings [68]. The intrinsic immunostimulatory properties of the mRNA molecule and its capacity to serve also as an immunoadjuvant are considered to be crucial benefits for the use of vaccines. The IVT-mRNA cancer vaccines strategy aims to immunize patients with Tumor-Associated Antigens (TAAs), which are mostly produced from isolated tumor cells that have been removed from patients (autologous tumor cells). IVT-mRNA technology is being tested in various clinical studies for oncologic reasons, since it promotes balanced humoral and cellular immune responses in animal models [68]. Prophylactic cancer vaccines are still a possibility. IVT-mRNA vaccines for cancer and IVT-mRNA COVID-19 vaccines share the commonality of recruiting IVT-mRNA technology to stimulate the immune system, but they have key differences, as shown in Table 1. 

TAAs and TSAs are the two main categories of tumor antigens. TAAs can be further subdivided into overexpressed antigens, cancer testis antigens, differentiation antigens, oncoviral antigens, and oncofetal antigens based on expression levels and tissue expression features. TAAs are commonly the target of cancer vaccines because they are overexpressed in cancer cells and may be common targets among individuals with the same malignancy. TAAs have been shown to be present in normal tissues as well, and vaccinations against them may cause central and peripheral tolerance reactions, resulting in poor vaccination efficacy or autoimmune disease against healthy tissues. Additionally, their poor distribution in vivo is a crucial factor in the failure of earlier cancer vaccines [76]. Finally, a major challenge is the suppressive tumor microenvironment, which also impedes T-cell infiltration into tumors and resulting in T-cell exhaustion. Therefore, it may be necessary to provide therapeutic vaccines with immune checkpoint inhibitors, as has been suggested for BNT111, a cancer vaccine combining four melanoma-associated antigens and a checkpoint inhibitor (NCT02410733) [77].

TSAs can be divided into two categories: those derived from abnormal regulation of gene expression and those derived from genetic mutations. Neoantigens, also known as TSAs or neoepitopes, are unique epitopes that are produced as a consequence of mutational alterations in the protein sequence and are not subject to central tolerance mechanisms. As a result, they may be used to develop personalized vaccines, coding for mutant neoantigens.

Advances in sequencing technology have not only sped up the collection of genomic and transcriptional data from cancer patients, but also exposed the enormous variety of disease-specific alterations in tumors. In fact, tumors from various patients share fewer than 5% of mutations. 

Therapeutic vaccines based on IVT-mRNAs have become a viable option for the exploitation of the potential of the patient-specific genomic data due to their simple design and scalable Good Manufacturing Practice (GMP) [78]. Particularly, IVT-mRNA stands out from other vaccines, such as those based on proteins or peptides, viral vectors, or DNA vaccines: IVT-mRNA has a strong safety profile, without the risk of infection or insertional mutagenesis since the vehicle is non-infectious and does not integrate into the host genome. IVT-mRNA is also only transiently available to transfected cells because of its sensitive nature and the harmful RNases, although by optimizing the molecule chemical composition and sequence, its lifespan may be adjusted as necessary [7].

Standard methods are used to develop IVT-mRNA cancer vaccines, which may be utilized to swiftly target patient-specific neoantigens found by investigating the tumor exome. The comprehensive identification and assessment of the neoantigens were only possible using massive parallel sequencing, which recognizes all coding alterations in malignancies. As tumor cells multiply, they develop mutations that give rise to distinct, new, or changed peptide sequences. The neoantigens are HLA-bound peptides that escape the central thymic tolerance, exhibit high immunogenicity because of their lack in normal tissues, and attract T-cells. Personalized IVT-mRNA mutanome vaccines enable the «mobilization of immunity» by setting the immune system up to target any subsequently emerging cancer cells, while protecting healthy tissues [79].

Compared to protein- and DC-based vaccinations, mRNA is unique for a number of reasons: (1) A complete protein containing MHCI and MHCII binding epitopes, or several antigens encoded simultaneously by IVT-mRNA, might support the humoral and cellular adaptive immune responses, resulting in heightened anti-tumor immunity; (2) IVT-mRNA vaccines have minimized insertional mutagenesis potential, when compared to DNA vaccinations, and are very degradable. The IVT-mRNA manufacturing process is devoid of harmful viral components and cellular elements, making it less susceptible to infection than protein- or cell-mediated vaccines. In current clinical trials, the majority of IVT-mRNA vaccines have been shown to be well tolerated, with very few instances of injection site responses; (3) The quick and scalable production of mRNA cancer vaccines is another benefit [80].

The personalized tumor mutanome «signature» of each patient may be quickly and affordably mapped, using high-throughput investigation of the genome and peptidome via advanced computational techniques and specific algorithms. Cancer patients undergo surgical resection of the tumor, followed by genomic or whole-exome sequencing and comparison of tumor- and normal-cell DNA from specific patients. Furthermore, RNA sequencing is conducted to confirm the actual expression of the identified variants within the tumor, and high throughput immunologic screening is used to find the specific mutations, leading to immunogenic neoantigens. Following the sequencing, up to 15–20 predicted neoantigens are properly selected, based on the sequencing procedure and their affinity to the patient’s HLA molecules, and they are subjected to computational prediction analysis. A customized therapeutic IVT-mRNA vaccine is designed and produced specifically for each patient and provided as a cGMP product. The mRNA vaccine is injected i.m. for four cycles every two weeks. As a result, the patient may develop T-cell responses to several neoantigen vaccines, exhibiting high levels of T-cell infiltration and targeting tumor cells that express certain neoantigens [81]. Blood samples will be obtained every two weeks (during the vaccination period) for circulating neoantigen-specific T-cells [82]. 

In this context, BioNTech (Mainz, Germany) has developed a number of clinical neoantigen vaccine candidates. Repeated injection of BNT121 was evaluated in a Phase II clinical trial for metastatic melanoma (NCT03815058), in combination with pembrolizumab, with strong immune responses. Currently, BNT122 (RO7198457), containing up to 20 unique patient neoepitopes, has an acceptable safety profile with mostly transient side effects [5]. BNT122 is also being evaluated in a Phase II for colorectal cancer (NCT04486378), pancreatic cancer research (NCT04161755), while a study for non-small cell lung cancer (NCT04267237) was withdrawn. Another personalized cancer vaccine by Moderna (Massachusetts, United States) mRNA-4157, exploiting LNPs, has the potential to include up to 34 neoantigens encoded on a single mRNA strand (also known as a neoantigen concatemer). Phase I and II clinical studies are taking place to evaluate the safety, tolerability, and immunogenicity of mRNA-4157, in combination with pembrolizumab, in participants with unresectable solid tumors (NCT03313778), and to assess its efficacy in participants with high-risk melanoma (NCT03897881).

## 4. Exploration of Cancer Immunotherapy: From mAbs to Adoptive Cell Therapy

Cancer immunotherapy was originated by William B. Coley, an American bone surgeon and cancer researcher, now known as the Father of Immunotherapy, who in 1893 used preparations of the bacterium *Streptococcus erysipelas* to inoculate them in situ in skin sarcomas, observing significant regression of tumors [83]. The fact that the immune system imposes continuous control over tumor initiation and promotion through tumor immune surveillance mechanisms was introduced in 1957 by Μ. Burnet [84]. One of these involves the recognition of MHC-presented TAAs of malignant cells by CD8+T-cells [85,86]. However, cancer cells employ several ways to evade immune surveillance (such as T-cell tolerance and exhaustion, direct inhibition, limitation of growth factors of immune cells, and restriction of immunogenic antigens) and build their tumor microenvironment [87]. 

Modern cancer immunotherapy includes a wide umbrella of therapeutics aimed at enhancing and/or altering the immune response of the organism to potentially treat cancer. The types of cancer immunotherapy are: (a) the cancer vaccines (analyzed in Section 3); (b) the Immune System Modulators; (c) the monoclonal antibodies (mAbs); (d) the Immune Checkpoints Inhibitors; (e) the T-cell transfer therapy; and (f) the Oncolytic Viruses.

The Immune System Modulators are a class of compounds or proteins able to enhance the immune system’s ability. The cytokines used in the clinical practice of tumor therapeutics are Interferon alfa (IFNα), Interleukin-2 (IL-2), and Granulocyte-Μacrophage Colony Stimulating Factor (GM-CSF), mainly acting as growth promoters of White Blood Cells [88,89]. The administration of Bacillus Calmette-Guérin (BCG) microbial preparation in advanced-stage bladder cancer patients yields encouraging results [90]. Moreover, micromolecular modulators of the immune system are already in clinical practice, such as lenalidomide, pomalidomide, and others. 

On the other hand, modern neoadjuvants are being exploited [87], such as the mAb-agonists of CD40 (selicrelumab) that lead to T-cell enrichment in the tumor area of pancreatic adenocarcinoma [91]. More than 100 mAbs have been approached for medical use until now, while those targeting cancer-specific antigens are a separate class of cancer immunotherapy drugs [92]. It is generally accepted that these mAbs act via more than one mechanism, involving the blocking of signals for proliferation, migration, and survival signaling. However, in most cases of IgG antibodies, the Antibody-Dependent Cellular Cytotoxicity (ADCC) and Complement-Dependent Cytotoxicity (CDC) are also involved [93]. Murine monoclonal antibodies were the first antibodies ever to be produced, and to reduce the undesired immune responses, chimeric, humanized, and finally, human mAbs have been constructed [94,95]. 

Anti-CD20 is the first monoclonal antibody approved by the FDA in 1997 for cancer immunotherapy (rituximab) for non-Hodgkin’s lymphoma [96]. Rituximab (IDEC-C2B8) is a chimeric monoclonal antibody produced in Chinese hamster ovary cells, containing human IgG1 heavy chains and mouse antigen-recognizing variable regions. Rituximab has direct anti-proliferative actions and promotes apoptosis in B lymphoma cells [97] via both ADCC and CDC mechanisms [98]. It is administered in relapsed, low grade or follicular non-Hodgkin’s lymphoma, alone or in combination with chemotherapy, with high response rates (>46%). Among its side effects is peripheral B cell depletion. Two versions of rituximab radio-conjugates were approved in early 2000: yttrium-90 Ibritumomab tiuxetan and iodine-131 tositumomab for non-Hodgkin’s lymphoma [99]. The Epidermal Growth Factor Receptor (EGFR) pathway is important in the body’s homeostasis [100]. It is over-activated in many cancer types, attributed to gene amplification, over-expression of ligands, and mutated pathway partners. Mutations in its extracellular region led to aberrant activation and increased proliferation and invasion. EGF serum levels are proposed as a prognostic marker for lymph node metastasis in head and neck cancer patients [101]. Inhibition by mAb includes cell growth inhibition via upregulation of cycle inhibitors (p27) in human prostate cancer cells [102,103]. Anti-EGFR mAbs have already been in clinical practice for many years in different malignancies (cetuximab and panitumumab for colorectal cancer, cetuximab for head and neck squamous cell carcinoma, and necitumumab for non-small cell cancer). Human epidermal growth factor receptor 2 (HER2/EGFR2) is a frequently overexpressed oncogene, mainly due to gene amplification, in both breast and ovarian cancer [104]. HER2 already resides in the clinical classification of breast cancer cases, and it is estimated that around 25% of these cases are HER2+ [105]. Transtuzumab was approved in 1998 in order to inhibit the ligand binding to HER2, yielding satisfactory results in both early and advanced metastatic breast cancer [106]. Pertuzumab, another anti-HER2 mAb, increases the overall survival of breast cancer patients from 40.8 to 50.6 months [107]. Tumor environment targeting mAbs, mainly anti-VEGF (Bevacizumab) and anti-VEGFR (Ramucirumab and Tanibirumab) are already approved for many types of cancer (colorectal, ovarian, renal, glioblastoma, and others) [108]. Major drawbacks are serious side-effects (stroke, proteinuria, bleeding, etc.), as well as the cancer cells’ resistance, through up-regulation of alternative angiogenic pathways. mAbs targeting alternative partners are both in development and clinical use. The field of the development of highly potent mAbs is emerging, and novel methodologies are being introduced. Among them are the bispecific antibodies (bsAbs), containing two separate scFvs. The Bispecific T-Cell Engagers (BiTEs) recognize a TAA in cancer cells and, in parallel, recruit T-cells in the tumor area to kill the recognized cancer cells through perforins and granzymes [109]. The most common BiTEs are designed to target CD3, such as Blinatumomab (CD3/CD19 BiTE), which is the first BiTE approved for relapsed or refractory B-ALL. Its administration is characterized by a 43% complete or partial remission and an increase in overall survival compared to conventional chemotherapy. The AMG420 BiTE (CD3/BSMA) succeeded in a 70% response rate in multiple melanoma patients (NCT02514239). 

Immune checkpoint inhibitors aim to dismiss the burdens of the immune system and enhance the anticancer response [110]. CTLA-4 (a Cytotoxic T Lymphocyte Antigen-4) is expressed after activation of T-cells (mainly the CD4+ T-cells) to replace a CD28 (a T-cell protein) in its interaction with the B7 family members on the surface of APCs, playing a critical co-stimulatory role in inducing T-cell activation. The interaction of CTLA-4 with the B7 leads to T-cell downregulation and functional inactivation. Furthermore, the PD1 (Programmed Death 1) protein at the cell surface of T-cells interacts with the PDL-1 (Programmed Death Ligand 1, belonging in the B7 family) on the surface of cancer cells, leading to T-cell exhaustion and their death. These two immune «brakes» were discovered by two independent research groups in the 1990′s by T. Honzo (for PD1) [111] and J.P. Allison (for CTLA-4) [112], who were awarded the Nobel prize in Physiology or Medicine in 2018. mAbs acting as PD-1 inhibitors (as Nivolumab), PDL-1 inhibitors (as Atezolimumab), and CTLA-4 inhibitors (as Ipilimumab) are routinely administered, and it is estimated that around 20–40% of cancer patients benefit from this administration. Molecular tests are proposed to be introduced, while studies with combinations of these antibodies and chemotherapeutics are already under way. Co-administration of anti-PD1 and anti-CTLA-4 mAbs to enhance effectiveness, has already been approved for metastatic melanoma, advanced renal carcinoma and metastatic colorectal cancer [113]. In addition, it is highly rationale to use a combination of chemotherapeutics and immune checkpoint inhibitors in order to increase the anti-tumor immune response since neoantigens are released due to tumor destruction. In 2017, the FDA approved the co-administration of pembrolizumab (anti-PD1 mAb) with pemetrexed and carboplatin [114].

T-cell transfer therapy, or adoptive cell therapy, is a highly promiscuous therapeutic approach based mainly on patients’ extracted T-cells, ex vivo selected, activated, and/or transduced, followed by proliferation and re-infusion back to the patient. There are two different approaches to T-cell transfer therapy: either exploiting the tumor infiltrating lymphocytes (TILs) or the adoptive T-cell Therapy (ACT) via the lentivirus transduced target specific TCR [115], as well as the Chimeric Antigen Receptor (CAR) T-cell therapy [116]. The latter is the topic of interest in the following sections. TILs (highly desirable are the CD8+), after their extraction from the patient’s tumor, are selected based on their ability to recognize the TAAs on the cancer cell surface being presented via the MHC class molecules [117]. The selected TILs are expanded in the lab and re-introduced into the patient, along with high quantities of IL-2 for their activation. In 1986, the co-administration of TILs with chemotherapeutics in mice was able to diminish metastatic tumors in organs such as the liver and lungs [118]. After that, the same group of Rosenberg et al. applied this protocol to 20 patients with metastatic melanoma and found that the administered TILs were able to halt tumor growth for 60% of them for up to two years [119]. Similar encouraging results have been obtained in other studies in melanoma [120], but also, via the proper selection of TILs, specific to TAAs, in cholangiocarcinoma [121], cervical cancer (for human papilloma virus-TILs) [122], ovarian cancer [123] and lung cancer [124]. The first TIL product is the Lifileucel (LN-144), intended for use in metastatic or unresectable melanoma and designated for duration in response for more than 2 years. 

Finally, Oncolytic Viruses (OVs) are an alternative approach, gaining worldwide interest in cancer immunotherapy. The idea behind this is to guide the virus via its intratumoral injection to destroy part of the tumor and then induce the inflammatory response in the area of the tumor via molecular recognition pattern release. This approach offers the advantage of targeted guidance of the virus and avoids the side effects of systemic administration. Talimogene Laherparepvec (T-VEC), a modified Herpes Simplex Virus (HSV) to express human GC-CSF, has been approved for certain types of melanoma (unresectable, metastatic) [125,126]. OVs armed into their genome with the scFv for a particular TAA is being tested as in the case of the recombinant HSV type 1, armed with the scFv against PD-1, in order for the OV to be guided in the tumor immunosuppressive microenvironment [127]. Increases in effector and memory CD8+ T lymphocytes were recorded in the tumor area of xenograft tumor mouse models.

## 5. Chimeric Antigen Receptor (CAR) T-Cells: Clinical Applications and Ongoing Trials in Cancer Immunotherapy

### 5.1. Clinical Implementation of CAR T-Cell Therapy

CAR T-cell therapies revolutionized the therapeutic options for patients suffering from hematological malignancies [128]. Ten years were celebrated in 2022, after the first clinical studies were conducted with CAR T-cells on leukemia patients [129]. The general concept behind this innovative therapy is the acquisition of autologous T-cells, through leukapheresis, to be virus-induced genetically modified to express the desired CAR receptor, and then to be expanded ex vivo. Until now, six (6) therapies have been approved by the FDA and EMA, targeting two TAAs, either CD19 or B-cell Maturation Antigen (BCMA), with the indication to treat hematological cancers. These therapies will be summarized thereafter. Patients receive the genetically modified CAR T-cells after lymphodepleting treatments (with cyclophosphamide and fludarabine) to increase the lifespan of the infused modified T-cells. Patients are hospitalized for several days thereafter to be monitored for emergent side effects.

CAR is a hybrid, artificial receptor that extracellularly has the scFv of a mAb, selected to recognize the desired TAA. In this way, a CAR receptor acts independently of MHC I or II antigen presentation, recognizing both processed and unprocessed epitopes, in contrast to the regular T-cell receptors (TCRs) [130]. Moreover, intracellular sequences, like the most common one, CD3ζ, and the co-stimulatory domains, CD28 or 4-1BB, guarantee the expansion and activation of T-cells. The transmembrane sequence (mostly the hinge region of IgG1) [131] secures the membranous localization of CAR. Upon cancer cell recognition, CAR T-cells act via multiple cytotoxic mechanisms, involving the release of perforins and granzymes, Fas ligand signaling, and secretion of inflammatory cytokines, such as the Tumor Necrosis Factor α (TNFα) and Interferon γ (IFNγ) [132]. 

CAR T therapy was developed in the late 1980’s by pioneering studies that deciphered the minimum sequences for constructing a CAR receptor, able to recognize the target epitope via antibody-targeted specificity and through its intracellular regions to elicit T-cell activation [130,133,134]. CD3ζ was determined as an independent signal to couple the necessary intracellular signal for T-cell activation [135,136]. Thereafter, significant output from ACT targeting cancer cells was gained by Carl June at the Perelman School of Medicine at the University of Pennsylvania. The production process was then established [137], and highly encouraging results were gained in pre-clinical studies with CAR T immunotherapy. Academia and industry (Novartis) began in 2012 the clinical studies with Tisagenlecleucel (CTL019), containing the scFv specific for CD19, along with CD3ζ and 4-1ΒΒ sequences [138]. Treatment of 79 children with refractory to rest anticancer therapies and/or relapsed B-ALL with Tisagenlecleucel led to an 82% overall response rate (ORR) (complete response or response with incomplete hematologic recovery) (NCT02435849, ELIANA study), with 49% of the responders having at least five years’ relapse-free survival (RFS). In another parallel study, Tisagenlecleucel was administered to adults’ patients with diffuse large B-cell lymphoma (NCT02445248, JULIET) with 53% ORR and progression free survival at 2.9 months. The aforementioned clinical studies along with many more conducted, led Tisagenlecleucel (Kymriah^®^) to be the first CAR T therapy approved in 2017 for pediatric and young patients with refractory and/or relapsed (r/r) B-ALL, diffuse large B cell lymphoma, and follicular lymphoma [138]. It is estimated that Kymriah^®^ has been administered to at least 7000 patients worldwide until now, and real-world data have been collected all over, confirming the clinical studies’ data [139,140]. Even more interestingly, a CAR-modified CD4^+^ T-cell clone was detected in patients’ blood 10 years after their initial treatment with CTL019, living a relapse-free life [141]. In a recent review, CAR T-cell therapy is called a living drug that has come to reform modern therapeutics beyond even anticancer strategies [142]. 

Axicabtageneciloleucel (Yescarta^®^) (Kite Pharma Inc., Los Angeles, California, United States) was approved in the EU (2018) and by the FDA (2022) for use in patients with r/r B cell lymphomas [143]. The CAR construct again the scFv for CD19, consisting of the CD3ζ activation domain and a CD28 costimulatory domain, transduced by retrovirus to patients’ T-cells [144]. ZUMA-1 (NCT02348216) is the first large-cohort study with axicabtageneciloleucel, including 111 patients with refractory large B lymphoma. The patients received a target dose of 2 × 10^6^ CAR T-cells/kg, resulting in a 40% complete response [145]. Brexucabtageneautoleucel (Tecartus^®^) (Kite Pharma Inc., Los Angeles, California, United States) employs the same manufacturer as Yescarta^®^ and similar construction details. In this product, in the manufacturing process, the removal of malignant cells from the isolated T-cells has been introduced in order to decrease the ex vivo exhaustion of CAR-engineered T-cells [146]. Tecartus^®^ was approved in 2019 and received a conditional authorization, waiting until 2025 for safety data. It refers to adults’ patients with recurrent cancer after 2–3 therapies, suffering from either mantle cell lymphoma (a subtype of non-Hodgkins’s lymphoma) or B-ALL. KTE-X19 or Brexucabtageneautoleucel performed a 56% complete response (NCT02614066, ZUMA-3), and long-term safety-associated data are waiting for authorization [147]. Idecabtagenevicleucel (Abecma^®^) (Bristol Myers Squibb, Tokyo, Japan) gained conditional authorization valid through August 2021. This product contains patients’ own lymphocytes engineered with a CAR receptor construct, employing the scFv for BCMA along with CD3ζ and 4-1BB costimulatory signals. BCMA is a promising TAA, already a marker for the identification of multiple myeloma cancer cells, since BCMA is present only in normal plasma cells [148,149,150]. The Idecabtagenevicleucel patients’ group concerns those with r/r multiple myeloma and is associated with 69% ORR, but it provokes severe cytokine release syndrome (at 94%), concluding by the authors that optimization in CAR T technologies is needed [151]. Ciltacabtageneautoleucel (Carvykti™) (Janssen Biotech, Inc., Titusville, New Jersey, United States) targets adults’ patients with r/r multiple myeloma who have followed three (3) previous treatments and failed (co-treatment with either an immunomodulatory agent, proteasome inhibitor, or anti-CD38 mAb). It is a unique product based on the generation of a CAR receptor with two (2) scFvs, targeting two different epitopes for BCMA (plus CD3ζ along with 4-1BB), thus performing with greater avidity. There is data about a superior performance to Abecma^®^, but further studies are needed (CARTITUDE-1, NCT03548207). Finally, lisocabtagenemaraleucel (Breyanzi^®^) (Bristol-Myers Squibb, Tokyo, Japan was authorized in 2022 to treat patients with r/r B cell lymphomas. This product offers the advantage of separating the CD4+ and CD8+ T-cells prior to their transduction with CAR (anti-CD19 CAR, CD3ζ, 4-1BB) [152]. The modified T-cells are administered separately (at a 1:1 ratio) to patients offering synergistic results, as proved in NOD/SCID/γc^−/−^ (NSG) mice [153]. Breyanzi^®^ achieved 53% complete response rates in patients [154].

CAR T technology has come to stay in modern therapeutics, and as such, any pitfalls related to side effects, medical feasibility issues, manufacturing, and cost have all to be assessed. Concerning the side effects of CAR T therapy, the most severe is Cytokine Release Syndrome (CRS), caused by the aberrant production of inflammatory molecules [marked by interleukin-6 (IL-6)] and observed in the first days of drug administration. As CRS can turn into a life-threatening condition, every hospital that administers a CAR T product has to be equipped with tocilizumab (an IL-6 receptor antagonist). Mice models partially failed to recapitulate this particular side effect, and medical and scientific communities should face this fact [155]. Among other side effects are allergic reactions, neurological problems, neutropenias associated with susceptibility to infections, and hypogammaglobinemia [138,156]. Although the risk of CAR T therapy-induced oncogenesis is low, it still exists, and the patients receiving this therapy are evaluated on a long-term basis [138]. In addition, CAR T therapy implements a risk for cardiovascular complications [157]. CAR T therapies are provided in certified hospitals and centers throughout the world, employing personnel trained in CRS syndrome and able to fulfill the CAR T Risk Evaluation and Mitigation Strategy (REMS) management plan. There are step-by-step approaches for the assessment and management of toxicities, including the different grades (1-4) of the CRS [158].

### 5.2. Next-Generation and Alternative TAA-Targeting CAR T Therapies in Clinical Studies

As a result, researchers focus their attention on several directions: the enhancement of the efficacy of CAR T therapies, the reduction of off-target consequences and T-cell exhaustion, as well as overriding the antigen escape. There is a gradient of co-stimulatory signals added to CAR receptors in order to potentially increase the antitumor responses: 1st generation CAR harbors only the CD3ζ co-stimulatory signal, while the 2nd generation ones, like those of the six (6) CAR T products already approved by the regulatory mechanisms, employ a supplementary co-stimulatory signal (CD28 or 4-1BB). The 3rd generation of CAR takes advantage of the simultaneous presence of multiple co-stimulatory signals (CD28, 4-1BB, CD27, OX40, and others) [159]. There are more than 1100 clinical studies [registered on the respective database of the National Institute of Health (NIH), https://clinicaltrials.gov/], assessed on 15 October 2023, under the term “CAR T-cells’’. Different strategies of CAR T constructs are employed in several types of cancer; around 100 of them have been recorded as completed, and indicative studies will be presented thereafter. 

T-cells of the 3rd generation of CAR for CD19 reached higher and more persistent levels in the plasma of non-Hodgkin’s lymphoma compared to the respective 2nd generation of CAR [160]. These classes of constructs undoubtedly need further research, specifically in larger cohorts of patients. Furthermore, there are a variety of next- generation CAR therapies, among them CAR T-cells redirected for universal cytokine-mediated killing (TRUCKs). In this approach, engineered CAR T-cells co-express cytokines (IL-7 and IL-18 supporting T-cell survival, and CCL19 acting as a chemo-attractant for mainly DCs) [129] (clinical results can be found at Table 2). The production of universal CAR T-cells via genetic engineering processes renders CAR T-cells capable of allogeneic use, which is undeniably a hallmark of the progress of this technology. Both CRISPR/Cas9 and transcription activator-like effector nuclease (TALEN) technology have already been employed to eliminate TCR and MHC in a first pilot study (NCT03399448). The feasibility of allogeneic administration in universal CAR T-cells was proven in the anti-NY-ESO-1 construct in B-ALL patients [161]. Another approach, designed to potentially halt the adverse side effects of CAR T therapy is the construction of switch adaptors (switchable CAR T-cells), employing drugs (like lenalidomide) as an on-switch of CAR activation [162], or UV exposure as an off-switch [163]. A construct of CAR, composed of an inducible form of caspase-9 by rimiducid, permits the induction of apoptosis of CAR T-cells, thus alleviating the CAR T-cells-induced side effects, e.g., the neurological syndrome [164]. Biphasic CAR (TanCAR) recognizes two different antigens, aiming to override antigen loss, frequently seen in highly proliferated cancer cells. TanCAR is designed in such a way that distinct activation of CAR T-cells occurs when one antigen of the pair exists, while a synergistic boost takes place when both of them are located in the tumor [165]. 

The success achieved by CAR T therapy in hematological malignancies is still waiting to be recapitulated in solid tumors [166]. Various parameters contribute to solid tumors’ resistance to CAR T therapy, and among them are: (a) the physical parameters, as solid tumors are frequently surrounded by scars of collagen; (b) the solid tumors’ heterogeneous population, thus expressing different antigens rendering difficult the identification of TAAs; (c) the impairment in homing of CAR T-cells in the anatomical spaces of solid tumors; as well as (d) the immunosuppressive tumor microenvironment. Some of the most frequent TAAs in CAR T therapy are mesothelin, Glypican-3 (GPC3), mucin-1 (MUC1), HER2, and EGFR, which are highly expressed in solid tumors [166]. The first such clinical trial was for patients with hepatocellular carcinoma with CAR T-cells targeting GPC3 [167]. In Table 2, indicative studies can be found for glioblastoma (where engraftment of CAR T clones in blood has been recorded for up to 29 months) as well as breast cancer. The results of the clinical studies of the next generation of CAR T therapies in solid tumors are anticipated to shed more light on the success stories in cancer therapeutics. 

**Table 2:** Indicative clinical studies of the different next generation CAR T therapies.

Code of CAR T Therapy	Construct	Malignancy	Transduction Method	Clinical Study-Result
Hematological Malignancies				
NCT01853631 [160]	CD19-CD3ζ-CD28-4-1BB (3rd generation)	non-Hodgkin’s lymphoma	retrovirus	Higher expansion and persistence of the 3rd generation CAR
NCT04381741 [168]	CD19-CD8- 4-1BB-CD3ζ-IL-7-CCL19 (TRUCK)	Large B cell lymphoma	lentivirus	ORR: 5/7 patients
NCT04557436 [169]	CD19 (Universal)	Pediatric, refractory B- ALL	lentivirus	Expansion of engineered CAR T-cells, but with serious side effects (Phase I)
NCT03016377 [164]	CD19-CD3ζ-4-1ΒΒ, Inducible caspase 9 (switchable)	Adult B-ALL	virus-induced	Improvement in CAR T therapy-induced side effects (Phase I)
NCT03233854 [170]	CD19vH-CD22vL-hinge- CD22vH-CD19vL-4-1BB-CD3ζ (Biphasic)	Adults B-ALL, Large B cell lymphoma	lentivirus	100% response with 88% CR (B-ALL) (Phase I)
Solid Tumors				
NCT03980288 [167]	GPC3-4-1BB-CD3ζ-Runt-related transcription factor 3 (RUNX3)	Hepatocellular carcinoma	lentivirus	Safety evaluation-Phase I
NCT02209376 [171]	EGFRvIII	Glioblastoma	lentivirus	Case report-prolongation in life expectancy in
NCT03740256	HER2 CAR T therapy and oncolytic virus	Breast Cancer	lentivirus	Recruiting
NCT05681650	HypoSti.CAR-HER2 T-cell therapy (Switchable)	Breast and other HER2+ Cancers	retrovirus	Not yet recruiting

## 6. Exploring the Synergy of CAR T-Cells and mRNA in Cancer Immunotherapy

As previously mentioned, most of the IVT-mRNA-based adoptive T-cell treatment has focused on producing CAR T-cells. CAR T-cell therapy generally entails the permanent genetic alteration of T-cells using viral vectors, such as retroviruses or lentiviruses, to introduce the CAR construct into the cells. In 1999, Clay et al. presented the first report on the successful transfer of a TCR into T-cells using a retroviral vector [172].

Several years later, in 2017, virally transduced CAR T-cells directed against CD19 were licensed by the FDA for the treatment of relapsed and refractory acute lymphoblastic leukemia and large B-cell lymphoma. While effective, concerns about off-target, on-target side effects, insertional mutagenesis, and issues managing CAR T-cell persistence in the body have inspired research on safer alternatives, with IVT-mRNA showing promising results and currently leading to numerous active clinical trials [173]. Furthermore, the capsid diameter of viral vectors (roughly 100 nm) limits their capacity to transduce lengthy gene cassettes, reducing transgene length up to 8–9 kb [174]. Additionally, the procedure of viral CAR T-cell engineering is carried out in GMP facilities, under biosafety level 2, takes time (2–3 weeks), requires skilled staff resources, hampering complexity in manufacturing, high costs, scarcity of manufacturing facilities around the world, and lot size restrictions [175]. Thus, genetic engineering technologies will need to face the above challenges and complexity while improving efficiency, safety, pricing and availability, cargo restrictions, and flexibility in the future.

In 2006, Zhao et al. [176], and Schaft et al. [177], were the first to describe the exploitation of IVT-mRNA to develop the approach of cancer T-cell receptor (TCR) immunotherapy, mainly using electroporation of primary T-cells, isolated from blood, with TCR targeting the gp100, resulting in functional CTLs against gp100+ melanoma cells. Additionally, from 2001 to 2005, it was shown that when IVT-mRNA was electroporated instead of DNA plasmids, DCs, and macrophages, CD40-activated B-cells and T-cells survivability increased (>80% viability) as well as transgenic expression efficiency (>90% efficiency) [176,178,179,180,181]. Those reports included the electroporation of TCRs against NY-ESO-1, MART-1, and p53 [173]. Pioneering immunotherapy research groups began to investigate an alternative strategy in 2009, employing IVT-mRNA to express CARs transiently in T-cells, ensuring high-quality and regulated generation of CAR-encoding mRNA against ErbB2, Her2/neu and carcinoembryonic antigen (CEA) [182,183]. In fact, IVT-mRNA-based CAR immunotherapy is a novel and quickly developing area of cancer therapy [184,185].

Recently, Reinhard et al. developed a strategy using the IVT-mRNA to direct CAR cells against the developmentally regulated tight junction protein claudin 6 (CLDN6) for treating CLDN6^+^ lung tumor without CRS that was effective at enhancing CAR-T cell persistence and in vivo proliferation [186]. Despite challenges such as the high tumor heterogeneity and the persistence of T-cell immune responses, the ongoing clinical trial (EudraCT No. 2019-004323-20) holds great promise for the future of CAR-T cell immunotherapy in head and neck cancer treatment.

Mesothelin is a highly selected target for CAR IVT-mRNA engineered cells, translated into the clinical phase [173]. In fact, all the completed clinical trials involving IVT-mRNA CAR cells were developed against mesothelin for malignant pleural mesothelioma (NCT01355965), metastatic pancreatic ductal adenocarcinoma (NCT01897415) and metastatic triple-negative breast cancer (NCT01837602). Ongoing/unknown status clinical trials explore the potential of the clinical translation of autologous T-cell IVT-mRNA engineered to express CARs against relapsed or refractory CD19^+^ leukemia and lymphoma (NCT03166878) and CD20^+^ B-cell malignancies (NCT02315118). Although no clinical updates have been given, a few additional clinical trials are still being conducted with individuals who have colorectal and breast cancer [187].

A recurrent requirement was the need for repeated dosing with 3–6 large doses, even though the studies reported that the adoptive IVT-mRNA CAR therapies were safe and typically free of major adverse events. In order to increase the duration of the in vivo activity in these individuals, numerous high-dose infusions of IVT-mRNA CAR T-cells are required, although repetitive dosing may result in severe side effects (e.g., severe anaphylactic shock [188]), due to the absence of genetically modified cell persistence [175].

IVT-mRNA CAR therapy shows a promising and safer therapeutic ability in hematologic and solid malignancies, according to a sizable amount of preclinical research [187]. However, additional preclinical and clinical research is needed to examine the efficiency in vivo and the intracellular delivery of the CAR IVT-mRNA, the cytotoxic and tumor-reducing efficacies of novel IVT-mRNA-engineered CAR cells against novel tumor antigens, inducing an effective antitumor response. Future research is necessary to overcome the IVT-mRNA’s lack of long-term activity and effectiveness. There is also a need for additional clinical trials to examine the therapeutic effectiveness of this alternative strategy. 

## 7. Recruitment of Our Novel, Patented Delivery Method of IVT-mRNAs via PTD Technology to Transduce CAR into NK-92 Cells

Current research on adoptive immunotherapy aims to potentiate efficacy, decrease the cost, and minimize severe side-effects, particularly those associated with CAR T therapy. Thus, the use of Natural Killer (NK) cells seems to be an attractive alternative to be exploited. CAR T-cells and CAR NK cells are both promising immunotherapies, but they differ in their cell origin, target antigens, antigen recognition and specificity, potential for GvHD, persistence, CRS generation, and manufacturing complexity. Table 3 summarizes the key differences between CAR T-cells and CAR NK cells. NK cells were discovered in 1975 by two independent research studies based on the ΝΚ cells ability to recognize and exert their cytolytic action on malignant, transformed cells [189,190]. NK cells are a major part of both innate and adaptive immunity, protecting against pathogens (mainly viruses, but also bacteria and fungi) through target recognition receptors [191]. Their killing mechanisms are mainly based on the granule exocytosis pathway, including perforins and granzymes that induce apoptotic cell death in target cells [192]. In addition, NK cells are indeed a major part of the immunosurveillance of cancer, as has been shown by numerous chemically induced tumors in mice, reviewed in a study by Markus et al. [193]. As a result, adoptive immunotherapy for equipping NK cells has gained worldwide interest [194]. To achieve an NK-dependent adoptive therapeutic approach, high quantities of NK cells are needed; however, they represent only a subpopulation of lymphocytes, thus restricting the ability for their acquisition from patients’ peripheral blood. Different sources of NK are being tested, such as umbilical cord, induced pluripotent stem cells (iPSCs), and stable cell lines [195]. NK-92 cells, the most commonly used NK cell line, derived from a non-Hodgkin lymphoma patient, are mainly characterized by a lack of allogeneic response [196,197]. NK-92 cells, as analyzed below, have already been administered to cancer patients, and must be irradiated before use in humans to inhibit their proliferation while at the same time maintaining their cytotoxic capability. Moreover, NK-92 cells are considered not to provoke GvHD, a major concern in CAR T therapy [198].

The first clinical trial with NK-92 cells in patients with either advanced renal carcinoma or melanoma concluded that their administration was well-tolerated, with mild side effects like fever, and there was a participant remaining alive 4 years later [199]. Moreover, it is highly important to mention that lymphodepleting chemotherapy, as in the case of CAR T-cell therapy, is not needed in NK-92 cells’ administration. Complementary, the infusion of a high number of cells (10^8^–10^10^/m^2^) to patients with both solid tumors and leukemia gave encouraging results, especially with the patients suffering from lung cancer [200]. The scale-up of production of NK-92 cells in bioreactors along with standardized manufacturing procedures helped a lot in the creation of «off-the-shelf» products. To increase their cytolytic performance, genetically engineered NK-92 cells to express Fc receptors (haNK^R^) have been constructed. These modified NK-92 cells are planned to be administered along with mAbs to enhance ADCC, as has already been done in the case of the anti-PDL-1 mAb [201].

Numerous preclinical studies highlight the success of CAR NK-92 or else targeted NK-92 (taNK) cells for treatment of hematological and solid tumors [202].CAR NK-92 cells targeted HER2 through lentivirus-mediated transduction, successfully restricting advanced neuroblastoma cells’ growth after their injection into the brains of mice [203]. The first clinical trial with lentivirus-transduced CAR NK-92 cells (anti-CD33, 3rd generation CAR) was conducted in 2018 with r/r AML patients [204]. This treatment caused mild side effects, and a complete hematological recovery, but it did not last. The short-term action of these engineered CAR NK-92 cells has been proposed by the authors as a cause of the irradiation, and the insertion of suicide genes should be exploited as an alternative to keep the functionality of NK-92 cells in vivo for as long as needed. Intracranial injection of anti-HER2 CAR NK-92 cells has been conducted recently in nine (9) patients with glioblastoma after their surgery, leading to disease stabilization in 55% of them (NCT03383978) [205]. Efforts have been made to enhance the durability and persistence of CAR NK-92 cells in tumor sites [194] via reprogramming the immune metabolism to halt functional exhaustion [202]. Currently, several clinical trials are underway by ImmunityBio Inc. (California, United States), the original proprietor of NK-92 cells and their variants, using non-viral methods of transfection, mainly electroporation, to transduce plasmids or IVT-mRNAs. In fact, the highly transducing property of NK-92 cells, in contrast to peripheral blood-derived NK cells, is another advantage offered by this cell line. Electroporation is correlated with high rates of cell death and a decrease in the functionality of the cells, while recently an efficient electroporation-based transduction protocol of plasmids for NK-92 cells has been presented by the group of L. Moretta [206]. The IVT-mRNA platform has the crucial advantage of facilitating adjustment to particular TAAs, among many other benefits, as analyzed in Section 6. Anti-CD19 CAR NK-92 cells were efficiently constructed via electroporation of the respective IVT-mRNA, targeting CLL cells, in culture [207]. 

In our recent work published in 2022, a novel technology has been presented based on PTDs for the transduction of the IVT-mRNAs for anti-ErbB CAR in NK-92 cells [50]. PTDs or CPPs are small-length peptides (usually less than 30 amino acids), with the prototype TAT basic domain (as PTD) derived from HIV-1, able to transverse cellular membranes and at the same time transfer many cargos [208]. PTDs have been exploited, via electrostatic self-assembly and no covalent conjugation, or fusion, as transporters inside cells of proteins, plasmids, siRNAs and IVT-mRNAs [27]. Over time, protein replacement therapy for monogenic/metabolic diseases [such as thalassemia and *SCO2* (for synthesis of cytochrome C oxidase) deficiency] has been successfully conducted by our research group [209,210,211,212]. In our patented chemical reaction(Greek patent, No: 1010063, titled «Method for the development of a delivery platform to produce deliverable PTD-IVT-mRNA therapeutics», with the International publication number: WO2021/094792 A1/20.05.2021, PCT/GR2020/000059), a peptide bond is being exploited to covalently bind the selected PTD (PFVYLI) to the IVT-mRNA of *SCO2* or *β-globin* for its intracellular delivery, leading to functional replacement of the respective proteins, in both *SCO2*-deficient, primary cells and bone marrow cells from β-thalassemic patients [49]. This novel transduction technology was employed again to transduce the IVT-mRNAs for two different, 2nd generation constructs of CAR (either with CD28 or 4-1BB as co-stimulatory sequences). It was found that the reaction of conjugation led to increased stabilization of the IVT-mRNAs and their significant protection from the action of RNases. The intracellular accumulation of the PTD-IVT-mRNAs of 2nd generation *CARs* rapidly accumulated within cells and reached their peak intracellular levels 24 h after transfection, suggesting their stability. Subsequently, their intracellular accumulation began to decline, but they remained detectable even at 120 h after transfection. The anti-ErbB-CAR-CD3ζ-(CD28 or 4-1BB) chimeric receptors were thus expressed at the expected molecular mass and possessed the correct membranous localization. At the same time, no negative impact on NK-92 cells’ morphology, growth, or viability was recorded to be caused by this transfection scheme. In our experiments, 2,3-butanediol was also used to enhance the cytotoxic potential of NK-92 cells by increasing perforin’s expression [213]. In the co-incubation experiments of the CAR-engineered NK-92 cells with two separate ErbB+ cancer cell lines, the human tongue squamous cell carcinoma (HSC-3), known to express ErbB receptors at quite high levels [214] and the breast adenocarcinoma MCF7 cell line, both constructs of the engineered 2nd generation CAR-NK-92 cells provoked cell death of target cells at high levels (at around 25%), in comparable ratios to lipofectamine-induced transduction of the corresponding IVT-mRNA of *CAR* (control of transfection). In fact, at the lower 5:1 (Effector: Target cells) ratio, a higher degree of cytotoxicity was assessed for the anti-ErbB-CAR-CD3ζ-4-1BB as compared to the respective chimeric receptor bearing the CD28 co-stimulatory domain sequence [50]. Moreover, MCF7 cells expressed only 25% of the ErbB2 protein levels compared to the HSC-3 cells. A possible explanation for this is that the TIE scFv (PanErbB) used in our constructs could also be tight to other ErbB receptors, such as homo- and heterodimers of ErbB1, ErbB2, and ErbB3 receptors, found in breast cancer cells [215]. 

## 8. Enhancing CAR NK Cell Potency: A Spotlight on Bioenergetics

A vital source of energy for living organisms is adenosine triphosphate (ATP), which is produced through the two major metabolic pathways, glycolysis and mitochondrial oxidative phosphorylation (OXPHOS). Cancer cells often use glycolysis to make ATP, even in the presence of oxygen, with the rate at which glucose is absorbed and preferentially producing lactate, via the well-known Warburg Effect [216]. Recent research, however, indicates that cancer cells’ ATP generation could switch to OXPHOS, if glycolysis is inhibited, thus improving mitochondrial function. It is anticipated that comprehending these events would lead to clarification of the mechanism of action of anticancer approaches and result in the development of effective therapeutics for cancer as well as neurological diseases and other disorders [217,218].

The influence of immune cell metabolism and bioenergetics (immunometabolism) on CAR cell cytotoxic capacity is a novel and emerging field in terms of improving the therapeutic outcome by regulating immunometabolism. In fact, it is well known that the cellular metabolism of immune cells substantially influences the immune response [219]. 

In macrophages, glycolysis has been associated with the conversion from the M2 (immune-suppressive) to the M1 (pro-inflammatory) phenotype. Also, in CAR T-cells, the presence of the co-stimulatory domains 4-1BB and CD28 was found to affect both their activation for tumor reduction and their survival for tumor elimination. These effects were attributed to changes in their metabolism [220]. The integration of signals through cytokine and germline-encoded activating and inhibitory receptors is necessary for NK cell activation in the context of tumors and viral infection, as are potential strategies used for «arming» NK cells for these environments, such as IL-15 priming. For NK effector functions like proliferation, killing, and the generation of interferon gamma (IFN-γ), metabolic fuels and pathways must be available [221]. 

Kawalekar et al. discovered notable variations in the metabolic and differentiation characteristics of CAR T-cells, employing CD28 or 4-1BB signaling regions. This study showed that T-cell metabolic reprogramming is flexible, and that the T-cells’ ultimate destiny may be affected by the CAR signaling domain. Aerobic glycolysis is the primary metabolic program in 28z CAR T-cells (including CD3ζ-CD28) [222], whereas oxidative metabolism, oxidative degradation of fatty acids, and mitochondrial biogenesis are the predominant metabolic programs in BBz CAR T-cells (CD3ζ-4-1BB) [223]. The increased survival and proliferative capacity of BBz versus 28z CAR-T-cells was aligned with the findings of several clinical studies [224]. The study also indicated that metabolic reprogramming of the CAR T-cells to promote either OXPHOS, which is characteristic of memory cells (BBz CAR-T-cells), or aerobic glycolysis, which is characteristic of effector cells (28z CAR-T-cells), may be one explanation for the differential persistence.

However, the characteristics of CAR co-stimulatory domains that affect persistence and resistance to exhaustion of CAR NK cells are still largely unknown, despite considerable clinical usage. Since NK cells are effectively exploited in cancer immunotherapy, several methods have been developed to take advantage of their capabilities. In this regard, immunometabolism has become an important topic, and NK cell metabolism is crucial to the control of the effector’s activities. Although the tumor microenvironment attempts to repress the metabolic activity of NK cells, metabolic limitation might be considerably improved by metabolic plasticity due to the co-stimulatory domains. The variations in metabolic setups among different types of NK cells remain unclear, underscoring the utmost importance of ongoing research in this area.

## 9. Challenges and Limitations of IVT-mRNA Cancer Immunotherapy Strategies

Cancer treatment strategies based on IVT-mRNA technology encompass a range of approaches, such as mRNA cancer vaccines, mRNA coding for cytokines [225], CAR-engineered cells, tumor suppressors, and various combination therapies. Despite their generally low occurrence, there is still a potential for minimal side effects. These side effects may be influenced by an individual’s genetic characteristics related to immune system reactivity. Variability in human immunity is attributed to genetic variations in genes responsible for TLRs, human leukocyte antigens (HLA), cytokines, and cytokine receptors [226]. One noteworthy adverse effect of SARS-CoV-2 IVT-mRNA vaccines associated with genetic factors is myocarditis [227]. Its occurrence is relatively rare, estimated at around 0.3 to 5.0 cases per 100,000 doses of COVID-19 mRNA vaccines, and in exceptionally rare instances, it can lead to fatalities. Recent findings have confirmed the involvement of genetic variants, potentially linked to HLA alleles, in myocarditis, particularly in monochorionic diamniotic twins [226,228]. Furthermore, it’s crucial to take into account potential adverse effects, like intense homeostatic proliferation and immune imprinting, during the design of mRNA vaccines [227].

However, the main current challenge in IVT-mRNA-based therapeutics lies in the improvement of stability [229] and delivery specificity to achieve systemic DCs targeting. Furthermore, there is a requirement for future investigations to address the challenges related to the sustained function and potency of IVT-mRNA. Foster et al. employed recent RNA technology to purify IVT-mRNA by incorporating a modified 1-methylpseudouridine nucleoside into the IVT-mRNA, thus minimizing the risk of immune stimulation. They also removed potential double-stranded RNA contaminants with the assistance of RNase III, which could impede translation [230]. Human CAR T-cells engineered with purified CD19 IVT-mRNA exhibited a two-fold increase in cytotoxicity against the Nalm-6 cell line and a remarkable 100-fold reduction in leukemia burden in humanized ALL mice, demonstrating enhanced persistence. Furthermore, additional animal studies should be designed to examine the optimal level of IVT-mRNA purity required before transfection and to investigate the potential of re-engineered mRNA in T-cells targeting tumor-specific antigens in conjunction with cytokine stimulatory signals [231]. These efforts have the potential to pave the way for an increased number of clinical trials involving IVT-mRNA CAR T therapy for hematologic and solid tumors in the future.

Achieving organ- or cell-selective mRNA delivery is the most important challenge in biomedical engineering and nanomedicine. Various lipid nanoparticles have been developed and optimized to increase cellular uptake and endosomal escape of IVT-mRNA-LNP formulations. Other lipid nanoparticles, such as antibody-conjugated LNPs and SORT LNPs, have been modulated to selectively accumulate in the target organs. Furthermore, hybrid nanoparticles containing polymers may facilitate the controlled release of IVT-mRNA. Other delivery strategies, such as the Selective Endogenous eNcapsidation for cellular Delivery (SEND), can also be applied for IVT-mRNA delivery [232]. Lastly, it is imperative to invest in research aimed at improving the homing of mRNA CAR T-cells within the tumor microenvironment by neutralizing localized immunosuppressive cues.

## 10. Future Directions in Cancer Immunotherapy: Paving the Way for Advancements

As tumors continuously evolve before and after treatment(s), both personalized pharmacogenomic analysis and post-therapeutics’ monitoring are needed to be integrated into modern medicine. The selection of the proper TAA(s) and/or TSAs is a key step in the successful outcome of CAR therapy. A reduction in EGFR expression was recorded in metastatic lesions of pancreatic cancer after CAR-T therapy [233]. Moreover, several anti-EGFR mAbs are ineffective in patients bearing tumors with *KRAS* and *NRAS* mutations. Molecular tests for EGFR and RAS are needed in clinical practice to offer individualized therapeutic approaches [234]. The development of personalized IVT-mRNA mutanome vaccines for cancer patients is one of the most groundbreaking highlights [235]. 

Universal, switchable, and Tan CAR and/or co-administrations with mAbs and OV, are among the highly promiscuous next-generation CAR. However, in most cases the cost of CAR T therapy reaches 250,000 $ to cover virus-induced transduction, cryopreservation, and manufacturing-related, multiple quality controls for each patient [197]. This high cost is estimated to be almost 10 times higher than the «off-the-shelf» CAR NK therapeutics. Current clinical trials with CAR NK cells employing the IVT-mRNA technology should be extended to large cancer patient cohorts to gain more meaningful results. The ultimate goal is to try to recapitulate the success recorded with CAR T cells in hematological malignancies, also in solid tumors, while at the same time reducing the severe side effects, cost, and complicated infrastructure needed.

## 11. Conclusions

The exploitation of the immune system to harness cancer has long been a hallmark of cancer research, exemplified by the numerous immunotherapeutic approaches [236]. CAR immunotherapy combined with IVT-mRNA technology has come to the limelight due to the effectiveness of CAR therapy against a variety of hematologic cancers and solid tumors and the boost given to mRNA technology due to COVID-19 vaccines. In the face of the difficulties brought about by the COVID-19 pandemic, mRNA vaccines have emerged to fulfill the need. IVT-mRNA-based technology can bridge a specific gap between protein therapy and gene therapy, having a variety of exceptional advantages over the other two macromolecule methods, such as rapid development, reduced manufacturing complexity, versatility, and mainly safety; there is no risk of insertional mutagenesis since for IVT-mRNA there is no requirement for nuclear entry.

In the early stages, IVT-mRNA faced limitations such as instability, high immunogenicity, and poor translatability. However, ongoing advancements in mRNA synthesis have significantly improved antigen expression and the development of protective immunity, while elevating the translation efficiency of IVT-mRNA by avoiding detection by RNA immune sensors. Advanced delivery methods, such as LNPs, have extended the intracellular lifespan of IVT-mRNA vaccines from mere minutes to weeks, enabling IVT-mRNA delivery to specific cell types, including T-cells [22,237,238].

IVT-mRNA technology is being explored and developed for cancer treatment applications, with several developments underway and exceptional clinical trials [50] being held for treating melanoma, squamous cell carcinoma, ovarian, breast, head and neck, prostate, pancreatic, metastatic renal, non-small cell lung cancer, and colorectal cancer. These vaccines include direct injection of IVT-mRNA into the tumor or surrounding tissue, as well as DC IVT-mRNA cancer vaccines, which entail the ex vivo loading of patient-derived DCs and represent the majority of mRNA cancer vaccines in clinical trials [60]. Strong in vivo antitumor T- or B-cell responses can also be produced by IVT-mRNA cancer vaccines when they are specifically tailored in accordance with the tumor antigens displayed by malignant cells, such as TAAs or TSAs/neoantigens [239]. Moreover, IVT-mRNA may encode cytokines that are considered to modulate the tumor microenvironment (IFNα2b, IL-2 and IL-12), inducing a broad immunity against cancer cells [240]. To strengthen the immune response to cancer cells, further strategies include combining IVT-mRNA with adjuvants, immune checkpoint inhibitors, gene editing instruments, or innovative delivery methods [241]. IVT-mRNA vaccines have the ability to encode whole proteins or numerous antigens at once, which may improve cellular and humoral immune responses, both of which are advantageous for protection against tumors. Unlike DNA vaccines, IVT-mRNA vaccines are degradable, do not integrate into DNA, and have a limited risk for insertional mutagenesis. Because there are no hazardous viral or cellular components in the production process, they are less prone to infection. In clinical studies, they are usually well tolerated with few injection site responses. Another benefit of mRNA cancer vaccines is their large-scale and rapid production.

In the context of immunotherapy, numerous preclinical and clinical studies on adoptive T-cell therapy for the treatment of cancer have yielded encouraging outcomes. The efficacy of the treatment regimens depends on the in vitro introduction of the TCR or CAR molecule into the host’s immune cells to direct them against cancer cells upon their re-infusion into the patient’s body. To date, several CAR T products or TCR-engineered T-cells have been approved by the FDA for the treatment of B-cell leukemia and lymphoma or have successfully entered clinical studies. Despite the high efficiency of CAR T immunotherapy in pediatric leukemias, this success unfortunately failed to be recapitulated in other target groups and/or types of cancer (older patients, solid tumors). Moreover, the severe toxicity associated with CAR T immunotherapy triggered researchers to search for adjustments and/or alternative approaches. The therapeutic effectiveness of CAR cells genetically manipulated with IVT-mRNA, although evaluated in a small number of clinical trials for the treatment of solid and hematologic malignancies, emerges IVT-mRNA as a game-changing and essential tool in the field of cancer immunotherapy. However, multiple infusions of IVT-mRNA- engineered CAR immune cells should also be taken into account.

Notably, our research group’s innovative PTD-IVT-mRNA delivery platform exemplifies the potential of delivering via the use of PTD technology, IVT-mRNA into NK-92 cells to engineer the CAR NK-92 cells, demonstrating that CAR T1E-engineered [242] NK-92 cells expressed the CAR construct safely. These CAR NK-92 cells demonstrated potent cytotoxicity against ErbB(+) cancer cell lines. Our work stands for the offer of a non-cytotoxic and costly-value alternative transduction technology to construct CAR NK-92 cells. Beyond the choice of CAR construct, the success of CAR NK-92 cell therapy hinges on other critical factors, including the choice of co-stimulatory signaling domains (e.g., CD28 and 4-1BB), shaping the metabolic profile (depending on glycolysis or OXPHOS) of CAR NK-92 cells, and influencing their effector functions. Understanding these metabolic differences could be crucial for optimizing CAR-NK cell immunotherapy in the context of cancer. By harnessing the metabolic plasticity imparted by co-stimulatory domains, we could potentially overcome the metabolic challenges posed by the tumor microenvironment. Further research is needed to explore variations in NK cell metabolism across different subtypes, emphasizing the importance of ongoing investigations in this field. 

Significant technological progress has raised the value of IVT-mRNA as a potent and adaptable cancer immunotherapy platform. Its effective advancement toward clinical translation, despite the formidable challenges, will significantly improve our capacity to fight cancer. Future research should keep concentrating on (but not limited to) comprehending and making use of IVT-mRNA’s paradoxical intrinsic innate immunity, enhancing antigen expression and presentation efficiency through the development of sophisticated and bearable delivery systems to achieve longer expression durations and effectiveness.

## Figures and Tables

**Table 1:** The differences between IVT-mRNA vaccines for cancer vs. IVT-mRNA COVID-19 vaccines.

	mRNA COVID-19 Vaccines	mRNA Vaccines for Cancer
Purpose	To trigger an immune response against a virus.	To stimulate the immune system to target and destroy cancer cells.
Antigen target	Spike protein of the SARS-CoV-2 virus.	Target tumor-specific/tumor-associated antigens unique to cancer cells or overexpressed in cancer antigens. Would be patient-specific or common among certain cancer types.
Immuneresponse	To generate neutralizing antibodies and activate the immune system to recognize and attack the SARS-CoV-2 virus.	To stimulate a robust T-cell-mediated immune response to target and eliminate cancer cells. Focus is on cytotoxic T cells.
Personalization	Not personalized. The same for everyone receiving the vaccine.	Could be designed to be personalized based on the patient’s specific tumor antigens (neoantigens), making them unique to each patient.
Immunogenicity	Spike protein is highly immunogenic vaccines induce a strong and rapid immune response.	Tumor antigens might not be very immunogenic, Additional strategies might be required to enhance their immunogenicity.
Clinical development	Been developed in a remarkably short timeframe due to the urgency of the COVID-19 pandemic. Enrollment of thousands healthy individuals in clinical trials.	Still in various stages of clinical development and face a longer and more complex path to approval, Clinical trials with a limited patient pool.
Manufacturing and distribution	Manufactured and distributed globally on a large scale to address the pandemic, They require distribution chains.	Manufacturing and distribution would be patient-specific or limited to specific cancer types, A different logistical challenge.
Clinical outcome	Significant efficacy in preventing COVID-19 infection and severe disease in large clinical trials.	Efficacy and clinical outcome vary by the type of cancer, patient-specific factors, and the stage of development.

**Table 3:** Key differences between CAR T-cells and CAR NK cells, as cell-based immunotherapies.

	CAR T-Cells	CAR NK-Cells
Cell Origin	- Usually derived from the patient’s own T-cells (autologous) - Universal T-cells via genetic engineering (in clinical trials)	- Derived from various sources, including patient, healthy donors, induced pluripotent stem cells (iPSCs), and cell lines - They are often allogeneic, making them an off-the-shelf treatment option
Target antigens	- Specific antigens expressed on the surface of cancer cells - Target antigen is predetermined - Usually, a protein associated with the cancer (TAAs/TSAs)	- NK cells have the inherent ability to recognize a broad spectrum of antigens on target cells - This makes them potentially suitable for a wider range of cancer types and other diseases
Specificity	- Highly specific to the chosen target antigen - They may not have the same natural ability to recognize and kill cancer cells as NK cells	- They combine the specificity of CARs with the natural cytotoxicity of NK cells, allowing them to target and kill cancer cells both specifically and through their innate mechanisms
Graft-vs-Host Disease (GvHD)	- Risk of GvHD when using allogeneic CAR T-cells, as they are derived from a donor and can recognize normal host tissues as foreign	-Less likely to cause GvHD due to their natural ability to distinguish between healthy and abnormal cells
Manufacturing Complexity	- Manufacturing could be complex and time-consuming, often requiring genetic modification, expansion, as well as selection of T-cells, cryopreservation, and transport facilities	- Manufacturing is generally simpler and faster, making them more accessible for patients
Cytokine Release Syndrome (CRS)	- CAR T-cell therapy is associated with a higher risk of CRS, a potentially severe immune reaction -Specialized personnel in hospital is needed to counteract the CRS	- CAR NK cells have a significant lower risk of causing CRS, which is a major advantage in terms of safety
Persistence	- CAR T-cells tend to persist in the body	- CAR NK cells may exhibit exhaustion - Trials to enhance their persistence via 3rd generation CAR NK cells and adjustment of their bioenergetics needs

## Data Availability

Not applicable.

## Abbreviations

ADCC	Antibody-Dependent Cellular Cytotoxicity
ALL	Acute Lymphocytic Leukemia
APCs	Antigen Presenting Cells
ARCAs	Anti-Reverse Cap Analogs
BCMA	B-cell Maturation Antigen
CAR	Chimeric Antigen Receptor
CDC	Complement-Dependent Cytotoxicity
CPPs	Cell Penetrating Peptides
CRS	Cytokine Release Syndrome
CTL	cytotoxic T-cell
DCs	Dendritic Cells
GvHD	Graft-versus-Host Disease
EGF	Epidermal Growth Factor
ErbB	Epidermal growth factor receptor
HER2	Human Epidermal growth factor receptor 2
HLA	Human Leukocyte Antigen
GMP	Good Manufacturing Practice
IFN	Interferon
iPSCs	induced Pluripotent Stem Cells
IVT-mRNAs	in vitro transcribed (IVT)-mRNAs
LNPs	Lipid Nanoparticles
mAbs	monoclonal antibodies
MHC	Major Histocompatibility Complex
NK cells	Natural Killer cells
OV	Oncolytic Viruses
PD1	Programmed Death 1
PTD	Protein Transduction Domain
r/r	refractory and/or relapsed
scFv	single chain Fragment variant
TAAs	Tumor-Associated Antigens
TCR	T-Cell Receptor
TILs	Tumor Infiltrating Lymphocytes
TNFα	Tumor Necrosis Factor α
TSAs	Tumor-Specific Antigens
UTRs	untranslated regions
Ψ	pseudouridine
